# A study of anxiety, depression and stress symptoms among Fayoum medical students during COVID-19 lockdown, Egypt

**DOI:** 10.1186/s41983-021-00377-2

**Published:** 2021-09-10

**Authors:** Mohamed R. Soltan, Shaimaa S. Soliman, Mariam E. Dawoud

**Affiliations:** 1 Psychiatry Department, Faculty of Medicine, Fayoum University, Faiyum, Egypt; 2 Public Health and Community Medicine Department, Faculty of Medicine, Menoufia University, Shibīn al-Kawm, Egypt; 3 Psychiatry Department, Faculty of Medicine, Fayoum University, POBox: 63514, Faiyum, Egypt

**Keywords:** COVID-19, Anxiety, Stress, Depression, Psychological impact

## Abstract

**Background:**

The widespread pandemic of COVID-19 virus carries not only physical hazards, but also major psychological effects especially among medical students. The aim was to investigate the psychological effects of COVID-19 on medical students and the factors affecting them. The study was carried out with an online questionnaire distributed through Google Forms for medical students at Faculty of Medicine, Egypt. The questionnaire included socio-demographic questions, validated psychometric tools for the assessment of depression, anxiety and stress (Depression Anxiety Stress Scales DASS-21) and the Impact of Event Stress Scale-Revised (IES-R) were applied.

**Results:**

The total number of participants was 282 students. Percentage of participants with clinically significant depression was 75.2%, anxiety was 56.4% and stress was 52.9%. Those showed PTSD probability was 54.3%. The multivariate regression analysis revealed that IES remained significantly associated with gender and previous road accidents, depression and anxiety remained significantly associated with personal history of psychiatric illness, while stress remained significantly associated with gender and previous road accidents.

**Conclusions:**

Medical students were highly depressed, anxious and stressed during period of COVID-19 pandemic. Being a female, having previous history of psychiatric illness and previous road accident were highly associated with increasing the psychological impact of COVID-19.

## Background

The novel coronavirus (COVID-2019) pandemic compromises international health concern which has spread very rapidly all over the world an outbreak of acute infectious pneumonia [3]. This large scale, infectious, public health event, had imposed enormous pressure on medical and healthcare providers, and the general public. The epidemic constitutes not only major physical health concern, but also unbearable psychological impact to people in the world [9, 12, 17].

The continuous spread of the pandemic, strict isolation measures and delays in starting schools, colleges, and universities across the country is expected to influence the mental health of college students. There are reports on the psychological impact of the epidemic on the general public, patients, medical staff, children, and older adults [6, 10, 19].

### Aim of the work

The aim of this work was to investigate the psychological distress of COVID-19 (depression, anxiety, stress, and impact of event related stress) on medical students, in Egypt, and to find out different personal risk factors which may provide a theoretical basis for psychological interventions to reduce their anticipatory suffering being in direct contact with infectious diseases.

## Methods

### Study population and sample

This is a cross-sectional study approved by the Research Ethics Committee and the Scientific Research Committee, Faculty of Medicine on April 2020. The target population comprised students of Faculty of Medicine, Fayoum University, from the 1st to 6th grade. Psychological impact of these students was assessed using online Google Forms containing structured questionnaire. This online form was available on the official Facebook group of the faculty through the period from 1st of May 2020 till the end of June 2020 and each participant can fill it in only once. The questionnaires were anonymous to ensure the confidentiality and reliability of the data. Finally, 282 respondents with valid completed questionnaires were included in the final analysis.

### Rating instruments

The study instrument comprised a structured questionnaire consisted of:

1. Socio-demographic information: gender, grade, past history of abuse, psychiatric disorders and family history of psychiatric disorders.
2. A well-validated psychometric tools for assessment of depression, anxiety and stress (Depression Anxiety Stress Scales DASS-21) and the Impact of Event Stress Scale-Revised (IES-R) in English version. The DASS [11] is a self- report scale which contains three subscales prepared to measure depression, anxiety and tension/stress. It is not categorical a categorical measure of clinical diagnoses. Each subscale contained 7 items. Participants were requested to use 4-point rated scales denoting severity to detect how far they have experienced each impact over the past week. Relevant items for depression, anxiety and stress were summed to calculate the scores. Impact of Event Scale-Revised (IES-R) [18] is a self-report containing 21 items denoting the experienced distress in response to traumatic events being living in the current pandemic situation also hearing news of deaths among medical professionals. Items correspond directly to 14 of the 17 DSM-IV symptoms of PTSD [13]. Respondents were asked to indicate the degree of distress during the past 7 days on each “difficulty” among the questionnaire list using 5-point scale ranging from 0 (“not at all”) to 4 (“extremely”) in response to COVID-19 as a stressful life event. Intrusion, Avoidance, and Hyperarousal subscales’ scores can be calculated separately [2].

### Statistical analysis of the collected data

An IBM compatible personal computer with SPSS statistical package version 23 was used to analyze Data (SPSS Inc., 2015. International Business Machines *Corporation* (*IBM*) SPSS statistics for windows, Armnok, NY, version 23.0). The variables were expressed in: number (No), percentage (%) mean (*x̅*) and standard deviation (SD). Association between qualitative variables was assessed using Chi-square test (*χ*^2^)*.* Fisher’s exact test was used in the case that any of the expected cells were less than five. A logistic regression was performed to ascertain the effects of possible risk factors on depression, anxiety and other outcomes. Two sided *p* value < 0.05 was considered statistically significant.

## Results

Two hundred eighty-two students participated in this survey. 64.2% were females, 81.6% did not have any history of road accidents, 58.5% did not have any history of psychiatric illness, 78.4% did not have any family history of psychiatric illness and 69.1% were worried from working in the medical field. Participants’ characteristics are detailed in Table 1.

**Table 1:** Participants’ characteristics (*n* = 282)

Character	No. (%)
Age in years (mean ± SD)	20.31 ± 1.61, 180.0–22.0
Gender	
Male	101 (35.8)
Female	181 (64.2)
Previous road accident	
No	230 (81.6)
Yes	52 (18.4)
History of psychiatric disorders	
No	165 (58.5)
Anxiety	59 (20.9)
Depression	41 (14.5)
OCD	14 (5.0)
Others	3 (1.1)
Family history of psychiatric disorders	
No	221 (78.4)
Anxiety	26 (9.2)
Depression	10 (3.5)
OCD	11 (3.9)
Others	14 (5.0)
Worry from working in the medical field in the COVID-19 era	
No	87 (30.8)
Yes	195 (69.1)

The mean score of the Impact of Event Scale-Revised total was 34.78 ± 13.94. The mean hyperarousal was 11.52 ± 5.19, the mean avoidance was 11.72 ± 5.90 and the mean intrusion was 11.53 ± 7.56. the studied group had mean depression of 21.97 ± 11.33, mean anxiety of 12.63 ± 10.38 and mean stress of 20.57 ± 11.36 (Table 2).

**Table 2:** Results of different scales among the studied group

Scale	Mean ± SD
Impact of Event Scale-Revised	
Total	34.78 ± 13.94
Hyperarousal	11.52 ± 5.19
Avoidance	11.72 ± 5.90
Intrusion	11.53 ± 7.56
DASS	
Depression	21.97 ± 11.33
Anxiety	12.63 ± 10.38
Stress	20.57 ± 11.36

The results of the Impact of Event Scale-Revised were as follows: no impact in 23.45%, clinical concern in 22.3%, probable PTSD in 9.6% and high PTSD enough to suppress immunity in 44.7%. Regarding the results of DASS; normal level of depressive symptoms was present in 11.7%, mild in 13.1%, moderate in 25.95, severe in 11.7% and extremely severe in 37.6%. Regarding anxiety symptoms, 37.95% was normal, but mild in 5.7%, moderate in 19.9%, severe in 11.05% and extremely severe in 25.5%. Normal stress level was present in 19.9%, mild in 27.3%, moderate in 22.0%, severe in 16.7% and extremely severe in 14.2% (Table 3).

**Table 3:** Prevalence of IES, depression, anxiety and stress symptoms among the studied groups

Scale	No. (%)
Impact of Event Scale-Revised	
No impact	66 (23.4)
Clinical concern	63 (22.3)
Probable PTSD	27 (9.6)
High PTSD enough to suppress immunity	126 (44.7)
Depression grade	
Normal	33 (11.7)
Mild	37 (13.1)
Moderate	73 (25.9)
Severe	33 (11.7)
Extremely severe	106 (37.6)
Anxiety grade	
Normal	107 (37.9)
Mild	16 (5.7)
Moderate	56 (19.9)
Severe	31 (11.0)
Extremely severe	72 (25.5)
Stress grade	
Normal	56 (19.9)
Mild	77 (27.3)
Moderate	62 (22.0)
Severe	47 (16.7)
Extremely severe	40 (14.2)

Abnormal IES was only significantly associated with female gender (*p* = 0.002). Any of the studied scales was not significantly associated with history of previous road accidents. Current depression was significantly associated with previous personal history of depression (*p* = 0.038) and current anxiety was significantly associated with personal history of anxiety (*p* < 0.001). Any current psychiatric disorders were not associated with family history of psychiatric disorders. This is detailed in Table 4.

**Table 4:** Association between possible risk factor and different scales

Character	IES (*n* = 216) No. (%)	Depression (*n* = 212)	Anxiety (*n* = 159)	Stress (*n* = 149)
Gender				
Male	67 (31.0)	72 (34.0)	52 (32.7)	46 (30.9)
Female	149 (69)*	140 (66.0)	107 (67.3)	103 (69.1)
*χ*^2^	9.23	1.27	1.53	3.35
*p* value	0.002^†^	0.259	0.215	0.067
Previous road accident				
No	172 (79.6)	174 (82.1)	126 (79.2)	116 (77.9)
Yes	44 (20.4)	38 (17.9)	33 (22.8)	33 (22.1)
*χ*^2^	2.28	0.15	1.29	2.88
*p* value	0.130	0.698	0.254	0.089
History of psychiatric disorders				
No	118 (54.6)	115 (54.2)	78 (49.1)	82 (55.0)
Anxiety	52 (24.1)	49 (23.1)	46 (28.9)*	34 (22.8)
Depression	33 (15.3)	36 (17.0)*	28 (17.6)	25 (16.8)
OCD	10 (4.6)	9 (4.2)	5 (3.1)	7 (4.7)
Others	3 (1.4)	3 (1.4)	2 (1.3)	1 (0.7)
FE test	7.97	9.60	22.18	2.88
*p* value	0.079	0.038^†^	< 0.001^†^	0.599
Family history of psychiatric disorders				
No	169 (78.2)	165 (77.8)	122 (76.7)	113 (75.8)
Anxiety	18 (8.3)	18 (8.5)	13 (8.2)	14 (9.4)
Depression	9 (4.2)	9 (4.2)	8 (5.0)	8 (5.4)
OCD	8 (3.7)	8 (3.8)	6 (3.8)	6 (4.0)
Others	12 (5.6)	12 (5.7)	10 (6.3)	8 (5.4)
FE test	2.29	2.28	4.00	3.31
*p* value	0.682	0.694	0.405	0.513

*χ*^2^: Chi-squared test, FE test: Fisher’s exact test

^†^*p* value is significant

^*^Significantly higher than their corresponding in the same variable

By univariate regression, the IES total score was significantly associated with gender, previous road accidents and personal history of psychiatric illness (*p* 0.022, 0.018 and 0.041, respectively). Depression was significantly associated with previous road accidents and personal history of psychiatric illness (*p* 0.038, < 0.001, respectively). Anxiety was significantly associated with gender and personal history of psychiatric illness (*p* 0.033 and 0.001, respectively), while stress was significantly associated with gender, previous road accidents, personal and family history of psychiatric illness (*p* 0.024, 0.003, 0.01 and 0.032, respectively) (Table 5).

**Table 5:** Univariate linear regression analysis

Character	IES *p* value	Depression *p* value	Anxiety *p* value	Stress *p* value
Gender	0.022	0.080	0.033	0.024
Previous road accident	0.018	0.038	0.188	0.003
History of psychiatric disorders	0.041	< 0.001	0.001	0.010
Family history of psychiatric disorders	0.014	0.195	0.157	0.032

The multivariate regression analysis of the significant univariate factors revealed that IES remained significantly associated with gender and previous road accidents (*p* 0.015 and 0.017, respectively), depression and anxiety remained significantly associated with personal history of psychiatric illness (*p* 0.001 for each) while stress remained significantly associated with gender and previous road accidents (*p* 0.011 and 0.004, respectively) (Table 6).

**Table 6:** Multivariate logistic regression analysis

Character	IES *p* value	Depression *p* value	Anxiety *p* value	Stress *p* value
Gender	0.015	–	0.036	0.011
Previous road accident	0.017	0.069	–	0.004
History of psychiatric disorders	0.070	0.001	0.001	0.050
Family history of psychiatric disorders	–	–	–	0.112

## Discussion

Medical students are particularly vulnerable to mental health concerns as a result of burden of their academic life and their job description requirements which increase their vulnerability to depression and anxiety. Hence the current pandemic adverse event may increase such negative feelings [7, 8, 20].

The findings of this study revealed a considerable negative impact on mental health in the studied group in which majority of the participants were experiencing increased stress and anxiety due to COVID-19 pandemic and lockdown.

In this study, according to DASS, those with clinically significant depression were 75.2%. The sum of clinically significant anxiety was 56.4%. Those with clinically significant stress were 52.9% (Table 3). The result of this study was in accordance with the results of a study held in Saudi Arabia where the majority of medical students (73%) were found to be stressed [1]. Our results were higher than the finding of a study held in India by Vala et al. [15] who used the same psychometric tool and found that depression, anxiety and stress were 15.6, 17.2, 10.8%, respectively. Yet, only 1st year medical students were assessed in the last study. Also, our results are higher than survey held in China among college students as all in which the overall incidence of anxiety was 26.60%. Depressive emotions were detected in 21.16% of the students [5]. Another Chinese study found that deteriorated mental health status among Chinese students [4]. Wang et al. [16, 17] found that in total, 53.8% of respondents rated the psychological impact of the outbreak as moderate or severe; 16.5% reported moderate to severe depressive symptoms; those who reported moderate to severe anxiety symptoms was 28.8%; while 8.1% of the respondents showed reported moderate to severe stress levels. This difference may be due to involvement of large sample among all college students not only medical students.

Regarding IES, the result of this study showed that 9.6% had the probability for PTSD while, 44.7% had high PTSD high enough to suppress immunity (Table 3), where females were significantly higher than male (Table 4). This was in line with a study done by Torun and Torun [14] in Turkey also, found that the average scores given to IES-R in women were also higher (*p* = 0.02).

Regarding association between different variables and psychological impact of COVID-19, the multivariate regression analysis of the significant univariate factors revealed that IES, depression, anxiety and stress are significantly related to gender, history of road accident and personal history of psychiatric disorders (Table 6). Studied variables such as being a female, student status, physical symptoms and poor self-rated health status were found to be significantly associated with a greater levels of stress, anxiety, and depression (*p* < 0.05) as a psychological impact of COVID-19. However, precise health information about treatment and local outbreak situation, particular precautionary measures were associated with and lower levels of stress, anxiety, and depression (*p* < 0.05) [16]. Also, the results of multivariate analysis in a study by Chang et al. [5], reported that those who live in rural areas, and reporting negative information regarding the epidemic were more likely to have anxiety. Yet, factors related to likeliness of depression were female gender, residence in suburbs, a drinking history, and excessive negative information concerning the epidemic.

Results of correlation analysis of psychological impact of COVID-19 assessed from Changzhi Medical College in China; indicated that economic effects, and effects on daily routine, and hanging in academic activities, were positively correlated with anxiety symptoms (*p* < 0.001). However, social support was negatively correlated with the level of anxiety (*p* < 0.001) [4]. Cao et al. [4] reported that living with parents was associated with significantly lower rates of severe anxiety in students, while living in rural areas, not having a steady income and recognizing somebody has the infection increased the risk of severe anxiety. These results concerning different studied variables may broaden the scope on the multifactorial nature of the psychological resilience which may affect psychological impact of different life stressors like the period of COVID-19.

## Conclusion

This study supported that medical students significantly affected with distress along the three axes of depression, anxiety and stress during the period of COVID-19 lockdown.

Being a female, having previous history of psychiatric illness and previous road accident were highly associated with increasing the psychological distress of COVID-19.

## Limitation of this study

The students were not motivated to fill in the form. Some recall bias about past traumatic events was not reported accurately by the students. Our research was done only in one medical school so the data cannot be generalized to all medical facilities in Egypt.

## Data Availability

Not applicable.

## Abbreviations

DASS-21: Depression Anxiety Stress Scales
IES-R: Impact of Event Stress Scale-Revised
PTSD: Post-traumatic stress disorder
